# MASH and the race for liver antifibrotics

**DOI:** 10.3389/fgstr.2025.1704078

**Published:** 2026-01-23

**Authors:** Quin Wills

**Affiliations:** Ochre Bio, Oxford, United Kingdom

**Keywords:** antifibrotics, combination therapies, liver fibrosis, metabolic dysfunction-associated steatohepatitis (MASH), metabolic therapies, precision medicine

## Abstract

Metabolic dysfunction-associated steatohepatitis (MASH), along with other chronic liver diseases, leads to progressive fibrosis and, ultimately, cirrhosis. Liver fibrosis is a major cause of global morbidity and mortality. Although past efforts to develop antifibrotic drugs have largely failed, recent advances in MASH metabolic therapies offer new hope. These include both indirect-acting agents such as glucagon-like peptide 1 (GLP-1) analogues, which reduce liver fat by promoting weight loss, and therapies with direct-acting mechanisms on the liver, such as thyroid hormone receptor beta (THRβ) activators and fibroblast growth factor 21 (FGF21) analogues. This perspective summarises emerging antifibrotics, from the fast-evolving class of metabolic therapies through to the more sluggish development of non-metabolic antifibrotics. We consider future therapeutic combinations and patient stratifiers that may impact patient outcomes, and close by asking if fibrosis reversal should be the only goal.

## 1 Introduction

Worldwide, 1/25 deaths is due to liver disease (1), with more than a quarter of the global adult population living with chronic liver disease, whether that be MASH, alcohol-associated liver disease (ALD), viral hepatitis, cholestatic disorders such as primary biliary cholangitis, or genetic disorders such as haemochromatosis. Of particular concern is the rising prevalence of the so-called MASH ‘tsunami’ (2), fuelled by rising societal obesity. The consequence of MASH and other chronic liver conditions is fibrosis – scarring that progresses to cirrhosis in its most severe form. To date, fibrosis remains the strongest predictor of liver-associated morbidity and mortality, and so its regression has become a therapeutic ‘holy grail’ (3).

**Figure 1:**
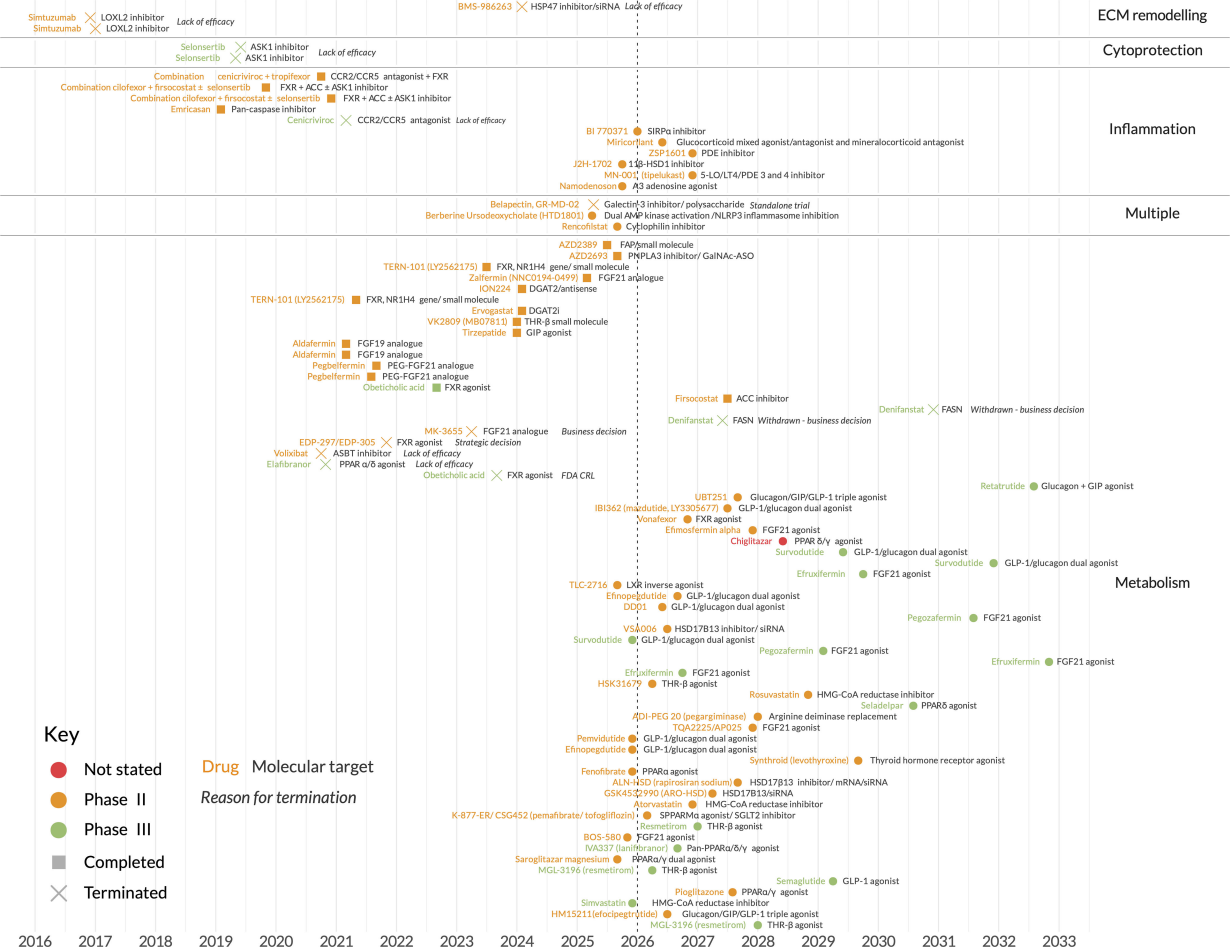
Key MASH clinical trials, past and ongoing. Dates shown are actual (past) or intended (future) completion dates. Further details are provided in **Supplementary Tables 1** and **2**.

Until recently, the search for agents that regress liver fibrosis has been met with repeated failure across multiple therapeutic classes (**Figure 1**, **Supplementary Tables 1** and **2**). Examples include (i) cytoprotectants such as apoptosis signal-regulating kinase 1 (ASK1) inhibitors and pan-caspase inhibitors (4, 5); (ii) metabolic therapies such as acetyl-CoA carboxylase (ACC) inhibitors and farnesoid X receptor (FXR) agonists (6, 7); (iii) chemokine inhibitors such as C-C chemokine receptor type 2/C-C chemokine receptor type 5 (CCR2/CCR5) inhibitors and galectin-3 inhibitors (8, 9); and (iv) extracellular matrix (ECM) remodellers such as lysyl oxidase homologue 2 (LOXL2) inhibitors (10). However, the field has now been energised with renewed optimism thanks to indirect- and direct-acting metabolic agents targeted at MASH patients.

## 2 MASH — fuelling an emerging metabolic toolbox

Metabolic therapies are shaping up as a critical success in chronic liver disease, with resmetirom (a direct-acting thyroid hormone receptor-beta [THR-β] agonist) becoming the first to receive US Food and Drug Administration (FDA) accelerated approval for non-cirrhotic MASH, in 2024. In a 52-week phase III trial (11), it achieved both histological surrogate endpoints required by the FDA and the European Medicines Agency (EMA) — improvement in steatohepatitis and fibrosis (12, 13). Twenty-six percent of patients achieved fibrosis improvement by at least one stage without worsening steatohepatitis, versus 14% with placebo (11). Although most patients still failed to reach a statistically significant response within 1 year, this has proven to be a seminal moment, with semaglutide (a glucagon-like peptide 1 [GLP-1] analogue) and efruxifermin (a fibroblast growth factor 21 [FGF21] analogue) rapidly following. In a 72-week phase III MASH trial, semaglutide improved fibrosis without worsening steatohepatitis in 37% of patients, versus 22% for placebo (14), resulting in its recent FDA approval (15).

While these trials have focused on pre-cirrhotic MASH, efruxifermin has become the first metabolic therapy to report positive results in more advanced fibrosis in a 96-week phase II trial of MASH patients with compensated cirrhosis (16). By intention-to-treat (ITT) analysis, 29% of patients demonstrated at least one stage improvement in liver fibrosis without steatohepatitis worsening, versus 11% with placebo (16). While a therapeutic intervention regressing cirrhosis may be paradigm shifting, the findings of histological improvement remain a surrogate endpoint. The true impact of these results remains to be seen in phase III trials and beyond, in which morbidity and mortality outcomes – such as the risk of hepatocellular carcinoma (HCC), decompensation, and transplant-free survival – will be the ultimate tests of efficacy (17).

With about 30 key trials expected to readout over the next 5 years (**Figure 1**, **Supplementary Tables 1** and **2**), metabolic therapies look set to become a frontline option for patients with MASH and compensated cirrhosis. How might this frontline evolve and be used? A key question of the toolbox is where direct-acting versus indirect-acting mechanisms may prove most useful. Therapies that do not promote weight loss may, for example, be preferred for the one in six patients with MASH classed as ‘lean MASH’ (those with a normal body mass index [BMI]), in whom the sarcopenic side-effect of GLP-1 analogues would be undesirable. On the other hand, GLP-1 analogues will likely be preferred as part of broader cardiometabolic interventions in patients with BMI >30 kg/m^2^ and comorbidities. Interestingly, the reduction in alcohol intake observed with semaglutide (18) may also enable a new therapeutic option to improve abstinence in ALD (alone or in combination with MASH [MET-ALD]) without the side-effects commonly experienced with disulfiram and naltrexone.

Beyond BMI and comorbidity considerations is the question of therapies with alternative metabolic mechanisms. With the current low therapeutic response rates it stands to reason that multiple metabolic mechanisms, with their respective addressable patient populations and side-effects, will prove valuable. Of particular interest is targeting the metabolism of other liver cell populations. The dominant mechanism of the therapies discussed thus far is understood to be largely via a relative increase in hepatocyte beta oxidation. However, denifanstat, a fatty acid synthase (FASN) inhibitor that reduces *de novo* lipogenesis in both hepatocytes and stellate cells, may become one such complementary approach. In the ITT analysis of its phase II trial, 30% of patients achieved at least one stage of fibrosis improvement, versus 14% with placebo – with subgroup analysis suggesting the greatest response in F3 patients (19). However, a planned phase III study has been paused prior to enrollment for business reasons. Another potential therapy known to directly inhibit stellate cell activation is lanifibranor. Interest in peroxisome proliferator-activated receptor (PPAR) agonists, such as lanifibranor, extends back several decades, despite weight gain and oedema limiting their use (20). Lanifibranor is currently in a phase III trial for MASH patients with type 2 diabetes (21), with pioglitazone (an insulin sensitising PPARγ agonist approved for type 2 diabetes) in multiple phase IV trials.

All things considered there is certainly promise for metabolic therapies of multiple mechanisms, but no clear home run yet beyond targeting weight loss, THRβ signalling, and FGF21 signalling.

## 3 An antifibrotic toolbox lacking diversity

While the low MASH therapy response rates are a crucial concern, another is that steatosis is not the primary driver of disease for a large proportion of liver fibrosis patients (22). This includes many patients in the later stages of MASH fibrosis. Globally, in 2017 hepatitis B (HBV), hepatitis C (HCV), and alcohol were responsible for most cases of compensated cirrhosis, 33%, 25%, and 21%, respectively, compared with 8% of cases due to MASH. Similarly, the major causes of death due to cirrhosis were HBV (29%), HCV (26%), and alcohol (25%), with only 9% due to MASH (22). So, it stands to further reason that broader, non-metabolic, antifibrotic mechanisms are required for widespread patient impact. Non-metabolic therapies such as LOXL2 and ASK1 inhibitors have been clinically tested as far back as a decade ago, with progress remaining slow.

Antibodies have proven enticing, despite the early failure of simtuzumab, which targets LOXL2, a collagen cross-linker (10). Nevertheless, current successes remain predominantly at phase I: (i) BI 765423, an interleukin (IL-11) inhibitor with antifibrotic and cytoprotective mechanisms (23) (ii) lixudebart, an anti-claudin-1 antibody that reduces cell adhesion and allows tissue remodelling (24), and (iii) BI 770371 which activates innate and adaptive anti-tumour immune responses also relevant to fibrosis (25), recently moving into phase II development for compensated cirrhosis due to MASH. Cell-based therapies have demonstrated promise, with a recent phase II study of autologous macrophage therapy in cirrhosis demonstrating improvement in transplant-free survival (26). A chimeric antigen receptor (CAR) T-cell therapy, initially developed to treat haematological malignancies (27), has also demonstrated the ability to clear senescent hepatic stellate cells (HSCs) (28–30).

As with biologics, small molecule and ribonucleic acid (RNA) therapies have yet to demonstrate clear successes. PLN-1474, a selective αvβ1 integrin inhibitor that reduces fibrosis via transforming growth factor beta (TGF-β) signalling, has reported favourable safety, a key concern with TGF-β inhibition (31). However, with intended co-development plans terminated, the next steps seem uncertain. BMS-986263, a small interfering RNA (siRNA) against collagen chaperone heat shock protein 47 (HSP47) has demonstrated early therapeutic promise in a phase II study of HCV with fibrosis (32, 33). However, a phase II trial in patients with compensated MASH cirrhosis was terminated for lack of efficacy (34). Selvigaltin (GB1211), a small molecule of the TGF-β signalling protein, galectin-3, has been well tolerated (35). However, further studies appear to have been halted due to a change in clinical development strategy (36).

Collectively, non-metabolic antifibrotics await their ‘resmetirom moment’ to provide the much-needed diversity to the antifibrotic toolbox. A toolbox that will likely consider therapies not only by mechanism, but their potency and tolerability as variables in a precision medicine endeavour.

## 4 Antifibrotic precision medicine — the right combination in the right patient, at the right time

Epidemiological studies suggest that variability in response to antifibrotic agents is to be expected. It has been long recognised that while some F3 fibrosis patients progress to cirrhosis within a few years, others remain stable for over a decade (37). Varied outcomes in metabolic patients are also well documented, with one study proposing an obesity group at increased risk of cardiovascular disease and type 2 diabetes, and a second with limited comorbidity risk despite similar liver steatosis levels (38). Intrapatient responses also vary over time, as observed in a cenicriviroc MASH phase II study where not all patients maintained a therapeutic response after 1 year (39).

Sex, ethnicity, and genetics provide further clues that the evolution of liver antifibrotics will have precision medicine at its core. Premenopausal women are less prone to MASH fibrosis and HCC (40, 41), although trials have yet to demonstrate clear sex differences. Individuals of Hispanic ancestry have a greater MASH prevalence and severity, partly due to the known patatin-like phospholipase domain-containing protein 3 (PNPLA3) gene risk allele, while individuals of some African ancestries exhibit lower liver steatosis and fibrosis (42). Population genetics provides further fascinating clues, with variants of two genes of particular MASH therapeutic interest (43). The PNPLA3 risk variant I148M is thought to reduce its hydrolyse activity and its degradation, resulting in increased triglycerides in hepatocytes and retinyl esters in stellate cells (43). The accumulation of poorly degraded, abnormal protein that sequesters cofactors of other lipases makes this a toxic gain-of-function that is amenable to inhibitors. AZD2693, a PNPLA3 antisense oligonucleotide in phase II (44, 45), and ALN-PNP, an siRNA in phase I (46), have been developed to reduce total PNPLA3, while ARO-PNPLA3, an siRNA (47) targets the variant. While PNPLA3 points to the metabolic roots of fibrosis, the mechanism of a second gene, HSD17B13, remains less clear. Its loss-of-function variants reduce inflammation without a reduction in steatosis (48). HSD17B13 therapies in phase II are GSK4532990 and ALN-HSD (separate licenses of the siRNA ARO-HSD) (49, 50), with INI-822, a small molecule inhibitor, in phase I (51).

While genetics, sex and ethnicity paint a picture of metabolic and non-metabolic stratifiers, perhaps the most challenging stratifiers will be age, comorbidities, and their interaction. Older patients live with a greater number of comorbidities, have a diminished liver regenerative capacity, and may not tolerate the effects of certain therapies (such as GLP-1 agonist-induced sarcopenia). The most common MASH comorbidities in older patients are, unsurprisingly, type 2 diabetes, dyslipidaemia, and hypertension. Diabetics, representing over half of the patients in the semaglutide phase III MASH trial (52), tend to have more significant fibrosis with microvascular damage, although response differences between those with or without diabetes are unclear. This may be a result of semaglutide’s dual therapeutic impact on diabetes and obesity. Notably, hypertension and dyslipidaemia have already emerged as a treatment stratifier, with the exclusion of obeticholic acid, an FXR agonist, due to its low-density lipoprotein-raising effect, unless the patient is on statins. Obeticholic acid has faced a particularly rocky path, with FDA restrictions on its use for primary biliary cholangitis, and denial of MASH approval (53).

For the clinician, the precision medicine mindset will mean balancing: (i) those therapies addressing the initial insult versus those with more general antifibrotic potential, and (ii) those therapies with high-potency, low-tolerability profiles versus those with greater tolerability but potentially lower potency. As of yet we can only speculate which patients will benefit first from a long-term therapy gradually addressing the initial insult, versus those in which a shorter induction phase with a potent, but less tolerable, antifibrotic would be best (54). What is clear is that few, if any, patients with more advanced disease will fully benefit from monotherapies. Sadly, past trials offer few lessons to guide future combination strategies (**Figure 1**, **Supplementary Tables 1** and **2**). Perhaps the most notable failure has been the MASH phase II combinations of selonsertib (an ASK 1 inhibitor), cilofexor (an FXR agonist), and firsocostat (an ACC inhibitor) (6). However, short study timelines (48 weeks) and inclusion of only the most significant fibrosis stages may partly explain the failure. In addition, these preclinical models and the proposed mechanistic rationales may be inadequate to elucidate the relevant outcomes. However, the reasons for the failure of these approaches remain unclear.

Despite limited data, the enthusiasm for augmenting GLP-1 therapies remains. Multi-agonist molecules being trialled include the glucose-dependent insulinotropic polypeptide (GIP) receptor agonist tirzepatide (55), the amylin receptor agonist amycretin (56), the glucagon receptor agonist survodutide (57), and the glucagon + GIP receptor agonist retatrutide (58, 59). Although some trials have focused solely on obesity, multi-agonism may improve antifibrotic potential. This is supported by survodutide’s FDA ‘breakthrough therapy’ designation for non-cirrhotic MASH (57), and tirzepatide’s effectiveness in resolving steatohepatitis without worsening of fibrosis (55).

## 5 Towards a fibrosis ‘cure’ and normal liver function

From MASH through to HCV infection, the extent of liver fibrosis has become established as the most meaningful surrogate endpoint for liver-associated morbidity (decompensation and HCC) and mortality (transplant-free survival) (3). Consequently, therapeutic reversal of fibrosis has become a central goal. As recently as 20 years ago, doubts remained as to how much reversal would be possible in significant liver fibrosis (‘F2’ portal fibrosis to ‘F3’ bridging fibrosis). However, the rising popularity in the early 2000s of bariatric surgery for obesity management, complete HBV suppression with tenofovir/entecavir, and the development of a cure for HCV by the mid-2010s, all helped set new expectations. Up to 33% reduction of fibrosis of at least one stage has been reported within 1 year after bariatric surgery (60), and a response of as much as 50% has been reported after 3–5 years in HCV patients with sustained virological response (61). More recently, FGF21 analogues have added to the enthusiasm for rapid and extensive fibrosis reversal, with efruxifermin, pegozafermin, and efimosfermin alpha phase II trials demonstrating remarkable response rates for significant fibrosis within 6 months (62–64). These results have elicited interest in acquisition of these compounds for potential phase III development, with efruxifermin currently under investigation for MASH in three phase III clinical trials (**Figure 1**, **Supplementary Table 1**).

Should full fibrosis reversal therefore be the sole therapeutic goal in MASH, especially in patients with cirrhosis? To date, the greatest body of evidence rests with HCV studies, which have demonstrated that while some complications such as raised portal pressure may resolve even in decompensated disease (65), HCC risk does not fully return to that expected of the fibrosis level the patient regresses to (66). Interestingly, an ongoing MASH phase III study of belapectin (a galectin-3 inhibitor) has also suggested that fibrosis and adverse outcomes may not be fully coupled (67). Analysis of the per-protocol patient population has indicated an 11–13% risk of oesophageal varices at 18 months (versus 22% with placebo) in the absence of fibrosis reversal (67).

MASH studies over the coming years will no doubt provide more insights. Until then, a reasonable assumption would be that tissue architecture and microcirculation disturbances, ongoing inflammatory processes (such as senescent hepatocytes) (68), and oncogenic changes that remain after fibrosis reversal will require a lifetime of patient surveillance. Equally plausible is that some patients with advanced disease will benefit from regenerative therapies aimed at restoring liver function and increasing transplant-free survival. Although regenerative therapies, such as mitogen-activated protein kinase kinase 4 (MKK4) inhibitors (69), have largely yet to progress beyond animal models, a phase II trial evaluating transplantation of hepatocytes into lymph nodes to supplement liver function (70) may prove another fascinating cellular therapy option. Perhaps as interesting will be if regenerative therapies themselves demonstrate antifibrotic potential, a response that is seen in patients whose HCV is cured. While the suppression of regeneration in fibrotic livers, via mechanisms such as TGF-β signalling (71), have long been recognised, recent work has further demonstrated that inhibition of microfibril-associated glycoprotein 4 (MFAP4), an ECM protein that interacts with integrins, both reduces fibrosis and increases regeneration in mice (72).

## 6 Conclusion

We are at the beginning of what can be expected to be a challenging phase in the development of a liver antifibrotic toolbox that adequately treats all patients across aetiology, stage of disease and across the many, as yet to be fully understood, stratifiers. Whether functional restoration is possible is still unclear. Nevertheless, we can celebrate the current successes with metabolic therapies that appear to reverse fibrosis in some MASH patients without worsening steatohepatitis. As we continue to push the therapeutic boundaries into cirrhosis, the value of reducing fibrosis as the sole therapeutic goal will also become clearer as long-term patient morbidity and mortality data become available over the coming decade.

## Data Availability

All relevant data is contained within the article: The original contributions presented in the review are included in the article/**Supplementary Material**.
