# Supplementary material for: MASH and the race for liver antifibrotics
Source: Front Gastroenterol (Lausanne). 2026 Jan 23;4:1704078. doi: 10.3389/fgstr.2025.1704078 (PMC12952438; doi:10.3389/fgstr.2025.1704078)
Supplement: Supplementary file 1 [file Table1.pdf]

Supplementary Material

1 Supplementary Tables

Supplementary Table 1: Key past, planned, and ongoing phase II and III trials of antifibrotic therapies in patients with significant fibrosis (F2/F3).

**Funding source**	**Drug name**	**Molecular target and/or mechanism**	**Condition**	**Clinical stage**	**Clinical trial identifier**	**Study start date**	**Completion date (actual or estimated)**
Gilead Sciences	Simtuzumab	LOXL2 inhibitor	MASH and stage 3-4 fibrosis (Ishak score)	Phase II	NCT01672866	Dec 2012	Dec 2016<br>Terminated
Gilead Sciences	Simtuzumab	LOXL2 inhibitor	MASH and stage ≥5 fibrosis (Ishak score)	Phase II	NCT01672879	Oct 2012	Jan 2017<br>Terminated
Allergan/AbbVie/ Tobira Therapeutics Inc.	Cenicriviroc	CCR2/CCR5 antagonist	MASH and F2/F3 fibrosis	Phase III	NCT03028740	Apr 2017	Mar 2021<br>Terminated
Gilead Sciences	Selonsertib	ASK1 inhibitor	MASH and F3 fibrosis	Phase III	NCT03053050	Feb 2017	Jun 2019<br>Terminated
Gilead Sciences	Combination cilofexor + firsocostat ± selonsertib	FXR + ACC ± ASK1 inhibitor	MASH or compensated cirrhosis with F3/F4 fibrosis	Phase II	NCT03449446	Mar 2018	Nov 2019<br>Completed
Gilead Sciences	Combination cilofexor + firsocostat ± selonsertib	FXR + ACC ± ASK1 inhibitor	MASLD or MASH with F3/F4 fibrosis	Phase II	NCT02781584	Jun 2016	Dec 2020<br>Completed
Novartis	Emricasan	Pan-caspase inhibitor	MASH and F1–F3 fibrosis	Phase IIb	NCT02686762	Jan 2016	Feb 2019<br>Completed

AbbVie/Novartis	Combination cenicriviroc + tropifexor	CCR2/CCR5 antagonist + FXR	MASH and F2/F3 fibrosis	Phase II	NCT03517540	Sep 2018	Oct 2020 Completed
Takeda (Shire)/Mirum Pharmaceuticals, Inc.	Volixibat	ASBT inhibitor	Mash and F0–3 fibrosis	Phase II	NCT02787304	Oct 2016	Oct 2020 Terminated
Genfit	Elafibranor	PPAR α/δ agonist	MASH and F1–F3 fibrosis	Phase III	NCT02704403	Mar 2016	Oct 2020 Terminated
Pfizer	Ervogastat	DGAT2i	MASH and F2/F3 fibrosis	Phase II	NCT04321031	Jun 2020	Feb 2024 Completed
Enanta	EDP-297/EDP-305	FXR agonist	MASH and F2/F3 fibrosis	Phase IIb	NCT04378010	Jan 2020	Nov 2021 Terminated
Bristol Myers Squibb	Pegbelfermin	PEG-FGF21 analogue	MASH and F3 fibrosis	Phase IIb	NCT03486899	Jun 2018	Aug 2021 Completed
NGM Biopharmaceuticals, Inc,	Aldafermin	FGF19 analogue	MASH and F2/F3 fibrosis	Phase IIb	NCT03912532	May 2019	Mar 2021 Completed
Intercept (alfasigma)	Obeticholic acid	FXR agonist	MASH and F2/F3 fibrosis	Phase III	NCT02548351	Sep 2015	Sep 2023 Terminated
Merck	MK-3655	FGF21 analogue	MASH and F2/F3 fibrosis	Phase IIb	NCT04583423	Jul 2020	Apr 2023 Terminated
Eli Lilly and Company	Tirzepatide	GIP agonist	MASH and F2/F3 fibrosis	Phase II	NCT04166773	Nov 2019	Jan 2024 Completed
Novo Nordisk	Zalfermin (NNC0194-0499)	FGF21 analogue	MASH and F2/F3 fibrosis	Phase IIb	NCT05016882	Aug 2021	Mar 2025 Completed

Sagimet Biosciences Inc.	Denifanstat	FASN	MASLD with MASH (fibrosis stage not specified, cirrhosis excluded)	Phase III	NCT06692283	Mar 2025	June 2027 Withdrawn
Sagimet Biosciences Inc.	Denifanstat	FASN	MASH and F2/F3 fibrosis	Phase III	NCT06594523	Mar 2025	Dec 2030 Withdrawn
Terns Pharma	TERN-101 (LY2562175)	FXR, NR1H4 gene/ small molecule	Non-cirrhotic presumed MASH (fibrosis stage not specified)	Phase II	NCT04328077	Jun 2020	May 2021 Completed
Terns Pharma	TERN-101 (LY2562175)	FXR, NR1H4 gene/ small molecule	Non-cirrhotic presumed MASH (fibrosis stage not specified)	Phase II	NCT05415722	Jun 2022	Jul 2023 Completed
Viking Therapeutics Inc.	VK2809 (MB07811)	THR-β small molecule	MASH Clinical Research Network (CRN) fibrosis stage 1–3	Phase II	NCT04173065	Nov 2019	Jan 2024 Completed
Ionis Pharma	ION224	DGAT2/antisense	MASH (fibrosis stage not specified)	Phase II	NCT04932512	Jun 2021	Feb 2024 Completed
HighTide Biopharma Pty Ltd	Berberine Ursodeoxycholate (HTD1801)	Dual AMP kinase activation /NLRP3 inflammasome inhibition	MASH with F2/F3 fibrosis, type 2 diabetes	Phase II	NCT05623189	Dec 2022	Apr 2025
AstraZeneca	AZD2389	FAP/small molecule	MASH or other steatotic liver disease with compensated fibrosis (stage not specified)	Phase II	NCT06750276	Dec 2024	Jul 2025 Completed
Gilead Sciences	Firsocostat	ACC inhibitor	MASH and non-cirrhotic fibrosis (stage not specified)	Phase II	NCT02856555	Aug 2016	Jul 2027 Completed
AstraZeneca	AZD2693	PNPLA3 inhibitor/ GalNAc-ASO	MASH and F2/F3 fibrosis in carriers of the PNPLA3 148M risk allele	Phase II	NCT05809934	Mar 2023	Sep 2025 Completed

Zydus Therapeutics, Inc.	Saroglitazar magnesium	PPARα/γ dual agonist	MASH and F2/F3 fibrosis	Phase II	NCT05011305	Aug 2021	Sep 2025
Hepion Pharmaceuticals, Inc.	Rencofilstat	Cyclophilin inhibitor	MASH and F2/F3 fibrosis	Phase II	NCT05402371	Oct 2022	Sep 2025
OrsoBio, Inc.	TLC-2716	LXR inverse agonist	Hypertriglyceridaemia and MASH/MASLD without cirrhosis	Phase II	NCT06564584	Aug 2024	Sep 2025
Can-Fite BioPharma	Namodenoson	A3 adenosine agonist	MASH and F1–F3 fibrosis	Phase II	NCT04697810	Dec 2021	Oct 2025
J2H Biotech	J2H-1702	11β-HSD1 inhibitor	MASH (fibrosis stage not specified)	Phase II	NCT06297434	Feb 2023	Oct 2025
Boston Pharmaceuticals	BOS-580	FGF21 agonist	Obese, MASH and F2 or F3 fibrosis	Phase II	NCT04880031	Sep 2021	Nov 2025
Boehringer Ingelheim	Survodutide	GLP-1/glucagon dual agonist	Obese/overweight, MASH without cirrhosis (fibrosis stage not specified)	Phase III	NCT06309992	Apr 2024	Dec 2025
Merck Sharp & Dohme LLC	Efinopegdutide	GLP-1/glucagon dual agonist	MASH and F2/F3 fibrosis	Phase II	NCT05877547	Jun 2023	Dec 2025
Altimmune, Inc.	Pemvidutide	GLP-1/glucagon dual agonist	MASH and F2/F3 fibrosis	Phase II	NCT05989711	Jul 2023	Dec 2025
Kowa Research Institute, Inc.	K-877-ER/ CSG452 (pemafibrate/ tofogliflozin)	SPPARMα agonist/ SGLT2 inhibitor	MASH and F1–F3 fibrosis	Phase II	NCT05327127	Nov 2022	Mar 2026
Haisco Pharmaceutical Group Co., Ltd	HSK31679	THR-β agonist	MASH and F2/F3 fibrosis	Phase II	NCT06168383	Nov 2023	Apr 2026
Madrigal Pharmaceuticals, Inc.	MGL-3196 (resmetirom)	THR-β agonist	MASLD and F1–F3 fibrosis or compensated MASH cirrhosis	Phase III	NCT04951219	Jul 2021	Apr 2026

Neuraly, Inc.	DD01	GLP-1/glucagon dual agonist	Overweight/obese, MASH/MASLD, and F1–F3 fibrosis	Phase II	NCT06410924	Jun 2024	Jun 2026
Hanmi Pharmaceutical Company Limited	HM15211 (efocipegtrutide)	Glucagon/GIP/GLP-1 triple agonist	MASH and F1–F3 fibrosis	Phase II	NCT04505436	Jul 2020	Jul 2026
Corcept Therapeutics	Miricorilant	Glucocorticoid mixed agonist/antagonist and mineralocorticoid antagonist	MASH and F1–F3 fibrosis	Phase II	NCT06108219	Oct 2023	Jun 2026
Visirna Therapeutics HK Limited	VSA006	HSD17B13 inhibitor/ siRNA	MASH and F2/F3 fibrosis	Phase II	NCT06322628	Apr 2024	Jul 2026
Inventiva Pharma	IVA337 (lanifibranor)	Pan-PPARα/δ/γ agonist	MASH and F2/F3 liver fibrosis	Phase III	NCT04849728	Aug 2021	Sep 2026
Akero Therapeutics, Inc	Efruxifermin	FGF21 agonist	Non-invasively diagnosed MASH/MASLD	Phase III	NCT06161571	Nov 2023	Oct 2026
Enyo Pharma	Vonafexor	FXR agonist	MASH and fibrosis (FIBROTEST score ≥ 0.28 and/or FIB-4 score ≥ 1.3)	Phase II	NCT06939816	Jul 2025	Nov 2026
MediciNova	MN-001 (tipelukast)	5-LO/LT4/PDE 3 and 4 inhibitor	MASLD, type 2 diabetes, and hypertriglyceridaemia without cirrhosis (advanced fibrosis excluded)	Phase II	NCT05464784	Aug 2022	Dec 2026
Mayo Clinic	Atorvastatin	HMG-CoA reductase inhibitor	MASH and F2/F3 liver fibrosis	Phase II	NCT04679376	Jan 2023	Dec 2026
Guangdong Raynovent Biotech Co., Ltd	ZSP1601	PDE inhibitor	MASH and F2/F3 fibrosis	Phase II	NCT05692492	Jun 2023	Dec 2026
GlaxoSmithKline	GSK4532990 (ARO-HSD)	HSD17B13/siRNA	MASH and F3/F4 fibrosis	Phase IIb	NCT05583344	Jan 2023	Apr 2027

Innovent Biologics (Suzhou) Co. Ltd	IBI362 (mazdutide, LY3305677)	GLP-1/glucagon dual agonist	MASH and F2/F3 fibrosis	Phase II	NCT06937749	Jul 2025	Jul 2027
University of Florida	Pioglitazone	PPARα/γ agonist	MASH and F1–F3 fibrosis	Phase II	NCT04501406	Dec 2020	Aug 2027
Regeneron Pharmaceuticals	ALN-HSD (rapirosiran sodium)	HSD17β13 inhibitor/ mRNA/siRNA	MASH and F2/F3 fibrosis with genetic risk	Phase II	NCT05519475	Feb 2023	Sep 2027
The United Bio-Technology (Hengqin) Co., Ltd.	UBT251	Glucagon/GIP/GLP-1 triple agonist	MASH and F2/F3 liver fibrosis	Phase II	NCT07145151	Aug 2025	Sep 2027
Chia Tai Tianqing Pharmaceutical Group Nanjing Shunxin Pharmaceutical Co., Ltd	TQA2225/AP025	FGF21 agonist	MASH and F1–F3 fibrosis	Phase II	NCT06569524	Sep 2023	Dec 2027
Madrigal Pharmaceuticals, Inc.	MGL-3196 (resmetirom)	THR-β agonist	MASH and F1–F3 fibrosis	Phase III	NCT03900429	Mar 2019	Jan 2028
Polaris Group	ADI-PEG 20 (pegargiminase)	Arginine deiminase replacement	MASH and F1–F3 fibrosis	Phase II	NCT05842512	Sep 2023	Jan 2028
89bio, Inc./Roche	Pegozafermin	FGF21 agonist	MASH and F2/F3 fibrosis	Phase III	NCT06318169	Mar 2024	Feb 2029
Novo Nordisk A/S	Semaglutide	GLP-1 agonist	MASH and F2/F3 liver fibrosis	Phase III	NCT04822181	Apr 2021	Apr 2029
VA Office of Research and Development	Synthroid (levothyroxine)	Thyroid hormone receptor agonist	MASH and F1–F3 liver fibrosis and type 2 diabetes, overweight/obese	Phase II	NCT05526144	Apr 2023	Sep 2029
Boehringer Ingelheim	Survodutide	GLP-1/glucagon dual agonist	MASH and F2/F3 fibrosis	Phase III	NCT06632444	Oct 2024	Dec 2031
Eli Lilly and Company	Retatrutide	Glucagon + GIP agonist	MASLD and at risk of developing major adverse liver outcomes	Phase III	NCT07165028	Oct 2025	Aug 2032

Akero Therapeutics, Inc.	Efruxifermin	FGF21 agonist	MASH and F2/F3 fibrosis	Phase III	NCT06215716	Dec 2023	Nov 2032

ACC, acetyl-CoA carboxylase; ADI, arginine deiminase; ALD, alcohol-associated liver disease; AMP, adenosine monophosphate; ASBT, apical sodium-dependent bile salt transporter; ASK, apoptosis signal-regulating kinase; ASO, antisense oligonucleotide; CCR, C-C chemokine receptor; DGAT; diacylglycerol o-acyltransferase homolog; FAP, fibroblast activation protein; FASN, fatty acid synthase; FGF21, fibroblast growth factor 21; FXR, farnesoid X receptor; GalNAc, N-acetyl galactosamine; GIP, gastric inhibitory polypeptide; GLP-1, glucagon- like peptide -1; HMG-CoA, 3-hydroxy-3-methylglutaryl-coenzyme A; HSD1, hydroxysteroid dehydrogenase type 1; HSD17β13 hydroxysteroid 17β dehydrogenase 13; LO, lipoxygenase; LOXL, lysyl oxidase homologue; LT, leukotriene; LXR, liver X receptor; MASH, metabolic dysfunction-associated steatohepatitis; MASLD, metabolic dysfunction-associated liver disease; mRNA, messenger RNA; NLRP3, NLR family pyrin domain containing 3; PDE, phosphodiesterase; PEG, pegylated arginine deiminase; PNPLA, patatin-like phospholipase domain-containing protein; PPAR, peroxisome proliferator-activated receptor; RNA, ribonucleic acid; SGLT2, sodium-glucose cotransporter 2; siRNA, small interfering RNA; SPPARMα, selective peroxisome proliferator-activated receptor α modulator; THR-β, thyroid hormone receptor-beta.
