# Supplementary material for: MASH and the race for liver antifibrotics
Source: Front Gastroenterol (Lausanne). 2026 Jan 23;4:1704078. doi: 10.3389/fgstr.2025.1704078 (PMC12952438; doi:10.3389/fgstr.2025.1704078)
Supplement: Supplementary file 2 [file Table2.pdf]

Supplementary Material

1 Supplementary Tables

Supplementary Table 2: Key past, planned, and ongoing phase II and III trials of antifibrotic therapies in MASH patients with compensated cirrhosis.

Funding source	Drug name	Molecular target and/or mechanism	Condition	Clinical stage	Clinical trial identifier	Study start date	Estimated completion date
Gilead Sciences	Selonsertib	ASK1 inhibitor	Compensated cirrhosis due MASH	Phase III	NCT03053063	Jan 2017	May 2019<br>Terminated
Bristol Myers Squibb	Pegbelfermin	PEG-FGF21 analogue	Compensated cirrhosis due to MASH	Phase IIb	NCT03486912	Jun 2018	Sep 2021<br>Completed
NGM Biopharmaceuticals, Inc,	Aldafermin	FGF19 analogue	Compensated cirrhosis due to MASH	Phase IIb	NCT04210245	Mar 2020	Feb 2023<br>Completed
Intercept Pharmaceuticals (Alfasigma)	Obeticholic acid	FXR agonist	Compensated cirrhosis due to MASH	Phase III	NCT03439254	Aug 2017	Sep 2022<br>Completed
Bristol-Myers Squibb	BMS-986263	HSP47 inhibitor/siRNA	Compensated cirrhosis due to MASH	Phase II	NCT04267393	Mar 2021	Feb 2024<br>Terminated
Galectin Therapeutics Inc	Belapectin, GR-MD-02	Galectin-3 inhibitor/ polysaccharide	NASH cirrhosis with portal hypertension but without oesophageal varices	Phase IIb/III	NCT04365868	Jun 2020	Apr 2025<br>Terminated
VA Office of Research and Development	Simvastatin	HMG-CoA reductase inhibitor	High-risk compensated cirrhosis	Phase III	NCT03654053	Oct 2020	Dec 2025

Xijing Hospital of Digestive Diseases	Fenofibrate	PPARα agonist	Compensated cirrhosis with primary biliary cholangitis	Phase II/III	NCT05749822	Feb 2023	Dec 2025
Boehringer Ingelheim	BI 770371	SIRPα inhibitor	Compensated cirrhosis due to MASH	Phase II	NCT06675929	Feb 2025	Jan 2026
Merck Sharp & Dohme LLC	Efinopegdutide	GLP-1/glucagon dual agonist	Compensated cirrhosis due to MASH	Phase II	NCT06465186	Jul 2024	Sep 2026
Madrigal Pharmaceuticals, Inc.	Resmetirom	THR-β agonist	Well-compensated MASH cirrhosis	Phase III	NCT05500222	Aug 2022	Jan 2027
Boston Pharmaceuticals/ GSK	Efimosfermin alpha	FGF21 agonist	Compensated cirrhosis due to MASH	Phase II	NCT06920043	Apr 2025	Dec 2027
Beijing Friendship Hospital	Chiglitazar	PPAR δ/γ agonist	MAFLD-related cirrhosis	Not stated	NCT06773221	Feb 2025	Jun 2028
National Institute of Diabetes and Digestive and Kidney Diseases (NIDDK)	Rosuvastatin	HMG-CoA reductase inhibitor	Compensated cirrhosis due to MASH, ALD, or chronic viral hepatitis	Phase II	NCT05832229	Oct 2023	Nov 2028
Boehringer Ingelheim	Survodutide	GLP-1/glucagon dual agonist	MASH with compensated cirrhosis	Phase III	NCT06632457	Dec 2024	Jun 2029
Akero Therapeutics, Inc.	Efruxifermin	FGF21 agonist	Compensated cirrhosis due to MASH	Phase III	NCT06528314	Sep 2024	Oct 2029
Gilead Sciences	Seladelpar	PPARδ agonist	Compensated cirrhosis with primary biliary cholangitis	Phase III	NCT06051617	Sep 2023	Aug 2030
89bio, Inc./Roche	Pegozafermin	FGF21 agonist	Compensated cirrhosis due to MASH	Phase III	NCT06419374	May 2024	Aug 2031

ASK, apoptosis signal-regulating kinase; ALD, alcohol-associated liver disease; FGF21, fibroblast growth factor 21; FXR, farnesoid X receptor; GLP-1, glucagon-like peptide 1; GR-MD-02, galactoarabinorhamnogalacturonate; HMG-CoA, 3-hydroxy-3-methylglutaryl-coenzyme A; HSP, heat shock protein; MAFLD, metabolic dysfunction-associated fatty liver disease; MASH, metabolic dysfunction-associated steatohepatitis; PEG-FGF, PEGylated fibroblast growth factor; PPAR, peroxisome proliferator-activated receptor; siRNA, small interfering RNA; SIRPα, signal regulatory protein alpha; THR-β, thyroid hormone receptor beta.
